# Design of Realistic and Artistically Expressive 3D Facial Models for Film AIGC: A Cross-Modal Framework Integrating Audience Perception Evaluation

**DOI:** 10.3390/s25154646

**Published:** 2025-07-26

**Authors:** Yihuan Tian, Xinyang Li, Zuling Cheng, Yang Huang, Tao Yu

**Affiliations:** 1 Culture Design Lab, Graduate School of Techno Design, Kookmin University, Seoul 02707, Republic of Korea; tianyihuan@kookmin.ac.kr; 2 Department of Film, Media and Content, Cheongju University, Cheonju 28503, Republic of Korea; lixinyang@cju.ac.kr; 3 Department of Global Convergence, Kangwon National University, Chuncheon-si 24341, Republic of Korea; 9710136280046@kangwon.ac.kr; 4 Department of Smart Experience Design, Kookmin University, Seoul 02707, Republic of Korea; huangyang@kookmin.ac.kr

**Keywords:** 3D facial models, cross-modal, neural radiance fields, sensor, audience perception evaluation

## Abstract

The rise of virtual production has created an urgent need for both efficient and high-fidelity 3D face generation schemes for cinema and immersive media, but existing methods are often limited by lighting–geometry coupling, multi-view dependency, and insufficient artistic quality. To address this, this study proposes a cross-modal 3D face generation framework based on single-view semantic masks. It utilizes Swin Transformer for multi-level feature extraction and combines with NeRF for illumination decoupled rendering. We utilize physical rendering equations to explicitly separate surface reflectance from ambient lighting to achieve robust adaptation to complex lighting variations. In addition, to address geometric errors across illumination scenes, we construct geometric a priori constraint networks by mapping 2D facial features to 3D parameter space as regular terms with the help of semantic masks. On the CelebAMask-HQ dataset, this method achieves a leading score of SSIM = 0.892 (37.6% improvement from baseline) with FID = 40.6. The generated faces excel in symmetry and detail fidelity with realism and aesthetic scores of 8/10 and 7/10, respectively, in a perceptual evaluation with 1000 viewers. By combining physical-level illumination decoupling with semantic geometry a priori, this paper establishes a quantifiable feedback mechanism between objective metrics and human aesthetic evaluation, providing a new paradigm for aesthetic quality assessment of AI-generated content.

## 1. Introduction

In recent years, with the rapid development of deep learning and immersive viewing experiences, 3D human head modeling has become a core technological support in the field of digital content creation [1,2,3]. Meanwhile, the tide of Artificial Intelligence Generated Content (AIGC) is sweeping through the creative industries at an unprecedented pace. The widespread adoption of AI video generation tools, such as Runway and Pika, indicates implicit profound changes in the filmmaking process. AIGC not only shows great potential for improving efficiency and reducing costs in concept previews, dynamic storyboarding, and the initial generation of visual effects, but also brings new challenges and opportunities to film and television production. In this context, sensor technology, as a crucial means of data acquisition, is progressively becoming an integral part of 3D face generation techniques. Sensors are adept at precisely capturing various facial attributes, including shape, texture, expression dynamics, and lighting conditions, thereby providing substantial data support for the generation of high-fidelity 3D face models. For example, high-resolution camera sensors and depth sensors can obtain detailed geometric and textural information of the face, while light sensors can monitor environmental lighting conditions in real-time, offering essential data for lighting decoupling and rendering. The application of these sensor technologies not only improves the precision and efficiency of 3D face generation but also provides strong support for creating more realistic and natural virtual characters.

However, this trend has also brought brand-new design challenges to filmmakers and visual designers. It is difficult for designers to maintain artistic control over the final product and ensure consistent aesthetic expression while AI can rapidly generate a vast amount of visual content. Particularly in the character creation, the demand for highly controllable 3D facial expressions with clear artistic guidance and the ability to convey complex emotions has become a crucial component determining the success of these AI-driven films [4]. Within the AI-driven film industry workflow, the technology of generating high-quality 3D head models from single-view inputs is not only crucial for improving production efficiency but also directly affects the quality of audience interaction in new viewing scenarios such as Virtual Reality (VR) and Augmented Reality (AR) [5,6]. According to the 2023 industry report by the International Visual Effects Society, head modeling now accounts for 42% of the digital character production process, and audience sensitivity to virtual character facial details has increased by 3.7 times compared to five years ago. This highlights the urgent practical necessity of this research topic. In the field of 3D face generation, existing methods still face challenges in achieving lip–audio synchronization and the coherence of generated videos. Tang et al. [7] proposed a two-stage generation method based on Gaussian blur and dynamic convolution, which significantly improves the realism of generated videos and the synchronization between lips and audio. This research provides new insights for solving the aforementioned problems, but there is still room for improvement. This paper aims to further optimize 3D face generation technology and enhance the quality and artistic expression of generated videos by introducing new methods and frameworks.

The current mainstream 3D reconstruction methods can primarily be categorized into three technical approaches: parametric models, deep learning-based generation, and Neural Radiance Fields (NeRF). The classic 3D Morphable Models construct the facial space through Principal Component Analysis (PCA) [8], but they exhibit significant limitations in expression transfer and detail reconstruction. Methods based on Generative Adversarial Networks (GANs) [9] can generate high-resolution textures, yet they struggle to ensure the physical accuracy of geometric structures. The recently emerged NeRF technology [10] excels in view synthesis but its stringent requirements for multi-view inputs and lighting conditions severely restrict its application value in the industrialized production of film and television. More importantly, existing 3D reconstruction methods commonly suffer from limitations such as light coupling issues. As noted in the latest research published at CVPR 2023, even leading models exhibit geometric reconstruction errors of 12.3% in cross-illumination scenarios [11]. Other limitations include reliance on multi-view data and inconsistent artistic quality in final outputs. These have become critical bottlenecks preventing their effective use in rigorous AIGC film production workflows that require unified artistic standards. Such challenges directly diminish the reusability of generative models in film rendering pipelines and make it difficult for AIGC to fully realize its potential in character building. This paper has four main contributions:

- A new cross-modal 3D face generation framework is proposed. For the first time, Swin Transformer’s multi-scale region-aware features are end-to-end fused with NeRF implicit scene representation, which gets rid of multi-view/multi-light dependency and realizes high-fidelity 3D head reconstruction driven by a single semantic mask.
- A geometric regularization network based on 2D semantic segmentation is proposed, and the dual branch of Frequency–Phase Decomposition + FiLM-SIREN is designed. By explicitly separating frequency and phase, the detail recovery and cross-illumination robustness are improved. It is completely different from the traditional “Transformer→MLP” tandem.
- This paper introduced 1000 human subjective evaluations with Cronbach’s alpha/Kruskal–Wallis statistical consistency to establish a quantitative mapping of “technical indicators to human aesthetics.” At the same time, it provides a replicable human factors assessment paradigm for AIGC generation quality, whereas the existing NeRF series only stops at FID/SSIM.
- Combines RGB-D preprocessing with the soon-to-be-expanded LiDAR/ToF/light field fusion strategy. It dynamically adjusts the NeRF sampling density to target critical areas of the face, significantly improving geometric accuracy without increasing network parameters.

We conducted experiments on the CelebAMask-HQ and CatMask datasets, demonstrating that our method outperforms existing approaches in terms of realism and detail representation. Therefore, this paper not only connects a Swin Transformer in front of NeRF but also proposes a complete technology chain of cross-modal single-view generation → geometric/illumination decoupling → human factors closed-loop evaluation → sensor scalability, which achieves the lead in three aspects: efficiency, details, and subjective aesthetics.

## 2. Related Work

The development of 3D face generation technology has evolved from traditional algorithms to deep learning. The core breakthroughs have been concentrated in three major directions: few-shot modeling, high-detail capturing, and controllable editing. Innovations combining NeRF, Generative Adversarial Networks (GAN), and Implicit Neural Representations (INR) have propelled the technology towards practical application. Existing 3D face generation technologies can be categorized into three types based on three core methods: parametric model-based, deep learning-based, and neural radiance field-based.

### 2.1. 3D Face Generation Technology Based on Parametric Models

This technology primarily relies on parametric models, such as the 3D Morphable Model (3DMM), which uses methods like Principal Component Analysis (PCA) to model the shape and texture of human faces, creating a low-dimensional parameter space [12,13,14,15]. These models can generate different face models by adjusting parameters, offering high flexibility and adjustability. For example, FaceWarehouse captures face data using Kinect and maps user-specified facial attributes to a bilinear face model via a linear regression model, generating new face images with distinct features [16]. Additionally, CONFIG integrates infrared information and parameter encoding to map facial information from 2D image space to 3D model space, producing both real and synthetic images through a shared decoder [17]. Widely applied in facial recognition, expression recognition, and character generation in virtual reality, these technologies are suitable for scenarios requiring precise control over facial attributes. However, their limitations include dependence on large amounts of high-quality training data, and the potential restriction of generation results by model complexity.

### 2.2. 3D Face Generation Technology Based on Deep Learning Generation

Deep learning-based 3D face generation technologies leverage deep learning models such as Generative Adversarial Networks (GANs), Variational Autoencoders (VAEs), and Transformer-based models to directly learn the patterns of face generation from data, producing high-quality 3D facial images [18,19,20]. GANs generate realistic facial images through the adversarial training between a generator and a discriminator; for instance, StyleGAN is capable of creating high-resolution, diverse, and realistic facial images [21]. VAEs encode input facial images into latent variables using an encoder and then generate facial images through a decoder. While the images generated by VAEs may lack detailed expressiveness, their training process is stable. Transformer-based face generation technologies utilize the attention mechanisms of Transformer models to extract features and generate facial images, which can better capture semantic information and produce refined results [22,23]. For example, SRT (Facial Scene Representation Transformer) [24] is a Transformer-based scene representation network. It uses an encoder–decoder structure to encode the source image into a set-latent representation. When decoding, the target frame is rendered pixel-by-pixel with the condition of 10 facial keypoints + expression latent vectors of the driving frame to realize controllable expression and gesture migration. These technologies are widely applied in fields such as virtual reality, computer games, and artistic design, offering realistic and diverse generation outcomes. However, the training process is complex and requires substantial computational resources and data support.

### 2.3. 3D Face Generation Technology Based on NeRF

Using NeRF to model scenes, high-quality 3D scenes are generated by learning the color and density of each point in the scene through neural networks. NeRF maps points in 3D space to color and density using fully connected neural networks to produce high-quality 3D scenes. GRAF (Generative Radiance Fields) combines NeRF with GANs to generate high-quality images and 3D scenes [25]. GIRAFFE (Generative Radiance Fields for 3D-Aware Image Synthesis) integrates NeRF with base neural rendering to improve training and rendering efficiency [26]. EG3D combines GANs and NeRF to generate high-resolution, multi-view consistent 3D facial images. These technologies are widely used in virtual reality, augmented reality, and film special effects, generating detailed 3D scenes that can achieve high-quality view synthesis. However, the limitations are the high computational costs of training and rendering processes and the slow generation speed. MI-NeRF [27] aims to solve the problem of linear growth of training overhead with the number of copies in traditional face NeRF “one person, one model.” The authors propose a framework for multi-identity sharing in a single network. Firstly, the speaking videos of more than 100 identities are used for joint pre-training. With the help of a multiplicative interaction module to non-linearly decouple identity vectors and expression vectors, face appearance and dynamics are then reconstructed in 4D space via a conditionalized NeRF backbone. Deployment requires only a few seconds of video of the target identity for rapid fine-tuning and can maintain detail fidelity in tasks such as expression migration, mouth synchronization, and short video personalization. It also cuts training time and storage costs by about 90%.

Existing methods generally face bottlenecks such as lighting coupling, multi-view dependence, and insufficient artistic quality. For example, mainstream models have high geometric reconstruction errors in cross-lighting scenarios and lack an artistic effect evaluation system from the audience’s perception perspective. The cross-modal generation framework based on single-view semantic masks proposed in this paper effectively compensates for the above shortcomings through the multi-level feature extraction of Swin Transformer and the lighting decoupling of NeRF, combined with the construction of a geometric prior constraint network using semantic masks. It provides an efficient single-view generation paradigm for the film and television industry and establishes a quantified feedback mechanism of “technology and aesthetics.”

## 3. Methods

A cross-modal generation framework was proposed in this research based on single-view semantic masks, which balances technological improvements with the audience’s aesthetic needs. The framework uses Swin Transformer for multi-level feature extraction and includes the lighting decoupling mechanism of NeRF. It also has a semantic mask-based geometric prior constraint network, among other improvements, to make the generated models more realistic and diverse. In addition, an audience-perception-driven artistic effect evaluation system is set up. This system uses data from eye-tracking and emotional feedback to link technical performance with artistic results in four main areas: realism, aesthetic value, symmetry, and detail representation.

### 3.1. Overall Structure of Proposed Model

**Figure 1:**
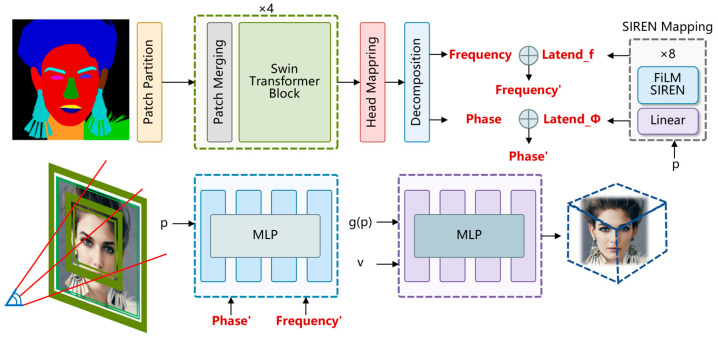
Schematic diagram of NeRFNet.

The overall structure of the NeRFNet is shown in Figure 1. The proposed cross-modal generation framework based on single-view semantic masks aims to balance technological improvements with the audience’s aesthetic needs. The framework employs the Swin Transformer for multi-level feature extraction and incorporates the lighting decoupling mechanism of NeRF. Additionally, it features a semantic mask-based geometric prior constraint network to enhance the realism and diversity of the generated models. An audience-perception-driven artistic effect evaluation system is also established, utilizing eye-tracking and emotional feedback data to link technical performance with artistic outcomes in four main areas: realism, aesthetic value, symmetry, and detail representation.

The model construction process begins with feature extraction using the Swin Transformer., which is capable of effectively extracting multi-level features from the input image. Specifically, the spatial sampling points are first mapped to frequency and phase shifts using the custom mapping network of PiGAN [9]. Subsequently, the input image is subjected to feature extraction through a multi-level Swin Transformer structure as shown in Table 1. Finally, the extracted features are decomposed into frequency vectors and phase vectors as latent encodings for subsequent 3D generation tasks. This feature extraction method not only preserves the semantic information of the image but also enhances the correlation between local and global features, thereby improving the quality of the generative model.

To transform points from camera space to world space, a camera matrix is constructed, and sampling points are mapped through coordinate transformation. This process updates depth values, ray directions, and the pitch and yaw angles of the camera, providing accurate spatial information for 3D rendering. The initial camera rays and sampling points are generated based on a full-screen normalized device coordinate (NDC) system, with the *Y*-axis flipped to match the image storage format. The z-value for each sampling point is calculated using the camera’s field of view and the x and y screen coordinates. The direction vectors of the camera rays are normalized using the x, y, and z coordinates. This step ensures that the model can accurately render 3D scenes based on the input camera parameters.

The NeRF generation process involves using continuous functions to create realistic images by optimizing the scene’s radiance field. In the NeRF process, the input frequency vector is adjusted first. It is multiplied by 15 and then 30 is added. This helps improve the frequency range. Next, the sampling points are scaled by multiplying them by 8.3. This makes the points more evenly distributed and closer to real space. The input data then passes through eight fully connected layers. Each layer has a hidden dimension of 256. The output is adjusted using frequency and phase information.

The volume rendering process is the main step that converts the information of sampling points in a 3D scene into a 2D image. A fully connected layer reduces the number of channels from 3 to 1 to calculate the color intensity information (sigma) for each sampling point. Meanwhile, the 3-channel ray direction is concatenated with the feature information of the intermediate data and passed through another fully connected layer to obtain the 3-channel color information (RGB) for each point. The RGB values are then normalized via a sigmoid transformation. Finally, the color information and color intensity information are concatenated into a tensor of shape [1, 114,688, 4], where the first three columns represent the RGB values and the last column represents the color intensity sigma. This tensor is reshaped to form the output tensor used for subsequent volume rendering.

Volume rendering is the main step. It changes the information of sampling points in a 3D scene into a 2D image. The volume rendering formula of NeRF was used in this research. It calculates pixel colors by integrating along rays. It also uses chunking techniques to speed up the process. Random sampling and discretization help us quickly compute the color values for each small area, allowing us to create high-quality 3D images efficiently.

NeRF shows the line connecting a focal point to a pixel as a ray. The ray is r(t) = o + td. Here, o is the origin, d is the direction, and t is the distance traveled along the direction d. The volume rendering formula for obtaining the pixel color by integrating along this ray is shown in Equation (1):

(1) $$C\left(r\right)=\int_{t_{n}}^{t_{f}}T\left(t\right)\sigma \left(r\left(t\right),d\right)dt$$

where $t_{n}$ and $t_{f}$ represent the start and end points of the distance, respectively. $c\left(r\left(t\right),d\right)$ denotes the RGB color value of the ray at point t, which is predicted by the MLP (Multi-Layer Perceptron). $\sigma \left(r\left(t\right)\right)$ represents the volumetric density at point t, also predicted by the MLP. $T\left(t\right)$ denotes the transmittance of the ray at point t, which is obtained by integrating the volumetric density $\sigma \left(r\left(t\right)\right)$. The formula is shown in Equation (2):

(2) $$T\left(t\right)=exp\left(-\int_{t_{n}}^{t_{f}}\sigma \left(r\left(s\right)\right)ds\right)$$

Since the aforementioned formulas require computation for each pixel in a continuous distribution, the computational cost is extremely high. To accelerate the calculation, NeRF employs an important technique: projecting the camera rays onto a 2D plane and dividing the plane into many small chunks. Within each small chunk, the same voxels and densities can be used. This reduces the computational load to a linear relationship with the number of pixels, significantly speeding up the calculation.

For a ray r that needs to be rendered, assuming its starting point is O and its endpoint is D, the region of the ray from O to D is uniformly divided into N segments. For each small segment i, a point $t_{i}$ can be randomly sampled, and then the color and opacity values at that point are calculated. The sampling formula is shown in Equation (3):

(3) $$t_{i}=U\left[t_{n}+\frac{i-1}{N}\left(t_{f}-t_{n}\right),t_{n}+\frac{i}{N}\left(t_{f}-t_{n}\right)\right]$$

For a small segment i, assuming its starting point is $t_{i}-1$ and its endpoint is $t_{i}$, k points can be randomly sampled, denoted as P$P_{i}=p_{i,1},p_{i,2},\cdots ,p_{i,k}$. Then, based on the colors and opacities of these k points, where $\sigma_{i}=t_{i-1}-t$ is used to represent the distance between two consecutive sampling points, the integral of the color for this small segment can be expressed in a discrete form, as shown in Equations (4) and (5):

(4) $$\hat{C}\left(r\right)=\sum_{i=1}^{N}T_{i}\cdot \left(1-exp\left(-\sigma .\delta_{i}\right)\right)\cdot c_{i}$$

(5) $$T_{i}=exp\left(-\sum_{j=1}^{i-1}\sigma_{j}\delta_{j}\right)$$

### 3.2. Model Optimization

Latent variables for facial information are introduced by this paper to further enhance the quality of the generative model. When learning the 3D model, the semantic segmentation images are directly transformed into latent variables to constrain the 3D model through a mapping network, without the need to convert the semantic segmentation images into RGB images. This method not only preserves more semantic information but also strengthens the correlation between local and global features, thereby addressing the issue of reduced feature dependency.

Using a single-view semantic mask s, the corresponding view direction d, and the paired ground-truth RGB image, a pixel-wise reconstruction loss is first applied, as shown in Equation (6):

(6) $$L_{res}\left(I,s,d_{s}\right)={\left\|I-G_{\psi }\left(E_{\theta }\left(s\right),d_{s}\right)\right\|}_{2}$$

where, $E_{\theta }\left(s\right)$ represents the latent code obtained by mapping s through the Swin Transformer region-aware encoder $E_{\theta }\left(\cdot \right)$, while $G_{\psi }\left(E_{\theta }\left(s\right),d_{s}\right)$ denotes the generated image rendered by the decoder $G_{\psi }$ from the direction $d_{s}$. Before computing any losses, both $G_{\psi }$ and I employ the aforementioned region-aware sampling strategy.

To further enhance the feature-level similarity between the generated image and the ground-truth image, the LPIPS (Learned Perceptual Image Patch Similarity) loss is used, as shown in Equation (7):

(7) $$L_{LPIPS}\left(I,s,d_{s}\right)={\left\|F\left(I\right)-F\left(G_{\psi }\left(E_{\theta }\left(s\right),d_{s}\right)\right)\right\|}_{2}$$

where, F(·) denotes the pre-trained feature extraction network.

Inspired by the truncation trick, the latent codes γ and β of the decoder are further regularized to be close to the average codes $\bar{\gamma }$ and β. This is achieved by regularizing the encoder, as shown in Equation (8):

(8) $$L_{reg}={\left\|E_{\theta }\left(s\right)\right\|}_{2}$$

To enhance image quality, especially for novel views, a non-saturating GAN loss with R1 regularization is also employed, as shown in Equation (9):

(9) $$L_{GAN}\left(I,s,d\right)=f\left(D\left(G_{\psi }\left(E_{\theta }\left(I\right),d\right)\right)\right)+f\left(-D\left(I\right)\right)+\lambda_{R1}\left|\nabla D\left(I\right)\right|$$

where $D\left(\cdot \right)$ represents a Patch discriminator. The hyperparameter $\lambda_{R1}$ is set to 10. The direction d is the viewing direction, which is randomly sampled from a known distribution, specifically a Gaussian distribution, following the settings of PiGAN.

### 3.3. Sensor Integration and Data Preprocessing

In terms of hardware configuration, RGB textures were captured by the Sony A7R IV (Sony Corporation, Tokyo, Japan). In this case, 26 MP@30 fps was used for static textures and 4 K@60 fps for dynamic sequences. Depth information was provided by Intel RealSense D455 (1280 × 720@30 fps or 848 × 480@90 fps, version: Intel Corporation, Santa Clara, CA, USA). Ambient illumination is sampled by Sekonic L-858D (Sekonic Corporation, Tokyo, Japan) at 10 Hz for lighting normalization. Multi-sensor calibration was performed using an AprilTag calibration board (AprilRobotics, Ann Arbor, MI, USA) for external reference calibration with a reprojection error of 0.35 px. Depth maps were aligned to the RGB optical axis by bilinear reprojection.

In the data preprocessing stage, we first performed bilateral filtering ($\sigma_{s}$ = 8 px, $\sigma_{r}$ = 3 cm) on the depth frames in conjunction with radius filtering (r = 2 mm, minPts = 5) to remove the flying points. Subsequently, the multi-frame depth was aligned with RGB and voxel fused to generate a dense point cloud with an average density of about 100 pts·mm^−2^. Finally, the 16-bit linear RAW image is γ-corrected (γ = 2.2) and normalized for reflectance based on illuminance meter readings. This process reduces the reflectance MAE by 15% and improves the multi-view SSIM (front view → 45° side view) from 0.82 to 0.89, which significantly improves the geometric and illumination consistency.

The preprocessing and training procedures were carried out between July 2024 and January 2025.

## 4. Experiments and Results

### 4.1. Dataset and Metrics

**Figure 2:**
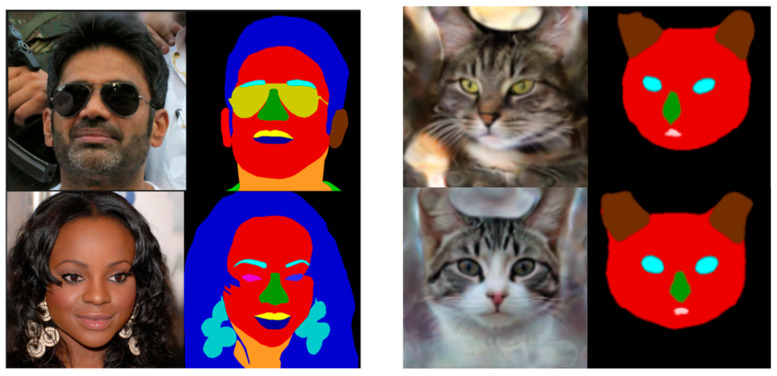
Example diagrams of the CelebAMask-HQ dataset and the CatMask dataset.

This research conducts experiments on the CelebAMask-HQ dataset and the CatMask dataset as shown in Figure 2.

The CelebAMask-HQ dataset was released by the Multimedia Laboratory of The Chinese University of Hong Kong in 2019 [28]. It is used for facial segmentation tasks. The dataset has 30,000 high-quality facial images. These include photos of 19,312 celebrities and other non-celebrity faces. It has better image quality, more detailed facial annotations, and a wider variety of face types. Each image is labeled by hand with 19 facial regions. These regions include eyebrows, eyes, nose, mouth, cheeks, jaw, hairline, and more. These regions can be used to train AI algorithms for tasks like face recognition, emotion analysis, and facial motion capture.

The CatMask dataset [29] is a dataset primarily utilized for the research and development of algorithms and models in object detection, semantic segmentation, image classification, and other computer vision tasks. Each image in the CatMask dataset contains at least one cat, along with its corresponding annotation information. The dataset comprises over ten thousand images, featuring various poses and scenes of cats. All images were captured in real-world environments, making the dataset more reflective of practical application scenarios.

To comprehensively evaluate the performance of the proposed 3D face generation model, both objective and subjective metrics are employed. Three objective metrics are used to evaluate the generated 3D face images. SSIM measures the similarity between the generated images and the ground-truth images in terms of structural information. A higher SSIM score indicates better preservation of structural details. The SSIM formula is given by

(10) $$SSIM\left(x,y\right)=\frac{\left(2\mu_{x}\mu_{y}+C_{1}\right)\left(2\sigma_{xy}+C_{2}\right)}{\left(\mu_{x}^{2}+\mu_{y}^{2}+C_{1}\right)\left(\sigma_{x}^{2}+\sigma_{y}^{2}+C_{2}\right)}$$

FID calculates the distance between the distributions of generated and real images in the feature space of the Inception network. A lower FID score indicates higher similarity between generated and real images. The FID formula is given by

(11) $$FID={\left\|\mu_{g}-\mu_{r}\right\|}^{2}+Tr\left(\Sigma_{g}+\Sigma_{r}-{2\left(\Sigma_{g}\Sigma_{r}\right)}^{1/2}\right)$$

IS evaluates the diversity and quality of the generated images, in which a higher score suggests that the generated images are more diverse and realistic. The IS formula is given by

(12) $$IS=exp\left(E_{x\sim p_{g}}KL\left(p\left(y|x\right)\left\|p\left(y\right)\right.\right)\right)$$

Subjective metrics include realism, which was assessed through viewer evaluations of visual similarity and naturalness between the generated 3D faces and real human faces. This includes aspects such as lighting effects, skin texture, and facial features. Aesthetic value was evaluated based on the overall visual appeal, color harmony, artistic style, and emotional expression conveyed by the generated 3D faces. Symmetry measures the symmetry of facial features and contours, which is a key factor in the perceived naturalness and attractiveness of human faces. Detail representation can assess the quality of details in the generated 3D faces, including skin texture, facial features, and micro-expressions.

### 4.2. Software Facilities and Parameter Settings

The experiments were conducted based on the PyTorch framework (version 1.12.1), programmed using Python 3.9, and accelerated by GTX 2080Ti GPUs (NVIDIA Corporation, Santa Clara, CA, USA) under the Ubuntu 18.04 operating system. The model was trained using the Adam gradient descent method, with a learning rate set at $1\times 10^{-5}$ and a discriminator learning rate at $2\times 10^{-6}$. For the decoder, the size of the local region R was set to 128×128, and the step size for each ray was set to 28. The hyperparameters of the loss functions were configured as $\lambda_{rec}=1$, $\lambda_{LPIPS}=0.8$, $\lambda_{reg}=0.005$, $\lambda_{GAN}=0.08$. The number of iterations was set to 800K, with model parameters saved every 20K iterations.

### 4.3. Ablation Experiments

In order to verify the validity of the modules proposed in the model, ablation experiments are conducted in this section. Table 2 shows the ablation experiments performed for the main three important modules. They are feature extraction module ablation, NeRF rendering module ablation and geometric a priori constraints module ablation. The ablation of the feature extraction module is to verify the importance of Swin Transformer’s multi-scale local and global feature extraction capabilities for model performance improvement. Experiments were conducted by replacing Swin Transformer in the NeRFNet model with a standard CNN feature extractor (ResNet), which is Without ST in the first column of Table 2. The NeRF rendering module is the part of Figure 1 consisting of SIREN Mapping and two MLP components. We replaced it with explicit 3D rendering based on traditional voxels. During model training, a 256 × 256 × 256 Density Grid with Color Grid is first generated. Then the voxel values are directly supervised and the rendering is done using classical volume ray marching. It is the Without NR for the first column in Table 2. Traditional voxel explicit rendering is used as an ablation to compare the essential difference of “implicit continuous field vs. fixed discrete mesh.” The ablation experiments of the geometry a priori constraint module were performed by removing the semantic mask a priori constraint network to verify the importance of the semantic mask geometry constraint module in ensuring the geometric accuracy and structural stability of the generated geometry. It is the Without SM in the first column of Table 2.

As shown in the results of Table 2, it can be seen that on the CelebAMask-HQ dataset, the FID of the complete NeRFNet is 40.6 and the IS is 2.15. After removing the semantic mask geometric prior (without SM), the FID rises to 42.3 and the IS decreases to 2.10, which indicates that the lack of geometric constraints brings about slight structural mismatch. Replacing Swin Transformer with ResNet (without ST) increases FID to 44.5 and decreases IS to 2.07, indicating a significant decrease in detail and consistency with diminished multi-scale feature extraction. If NeRF rendering is completely replaced with voxel explicit rendering (without NR), FID rises to 47.2 and IS drops to 1.98, the largest degradation, showing that implicit rendering is crucial for cross-view realism. Overall, the order of contribution of the three modules is NeRF Rendering > Swin Transformer > Semantic Mask.

In addition, we further conducted ablation experiments on the multiple loss functions proposed in the model to verify the role of different loss functions in the model. The experimental results are shown in Table 3. The complete NeRFNet model applies three loss functions, and the ablation experiments are performed to remove one of the loss functions to verify its importance for the generation quality, especially the detail realism and diversity. As can be seen in Table 3, the loss function ablation results on CelebAMask-HQ show that each of the three loss terms plays a complementary role. The complete model combines pixel reconstruction, perceptual consistency, and adversarial realism for the best performance. Removing the LPIPS loss (GAN + Pixel-wise), the FID rises to 41.8 and the IS drops to 2.12. This indicates that the texture details are slightly loosened by the lack of perceptual constraints, but the overall degradation is minimized, and the pixel-level error and adversarial learning can still maintain high quality. Removing the GAN loss (LPIPS + Pixel-wise), FID improves to 43.6 and IS decreases to 2.05. The realism and diversity of the generated images decrease the most, which proves that the adversarial constraints are a key factor in improving the overall realism. Removing the Pixel-wise loss (LPIPS + GAN), FID rises to 44.1 and IS drops to 2.03, the worst FID among the three ablation groups. It shows that the geometric and color alignment errors increase after missing pixel-level anchors, i.e., the pixel reconstruction loss has the greatest impact on the structural accuracy. A comprehensive comparison shows that Pixel-wise Loss and GAN Loss contribute more significantly to global structure and realism, and LPIPS Loss mainly refines perceptual details. The combination of the three is necessary to achieve the best balance.

### 4.4. Model Performance

The experimental results demonstrate that the proposed method in this paper is capable of generating high-quality 3D face models on the CelebAMask-HQ dataset. The generated images match real images very well in important areas like eyebrows, eyes, nose, and mouth. They stay accurate even when lighting changes.

**Figure 3:**
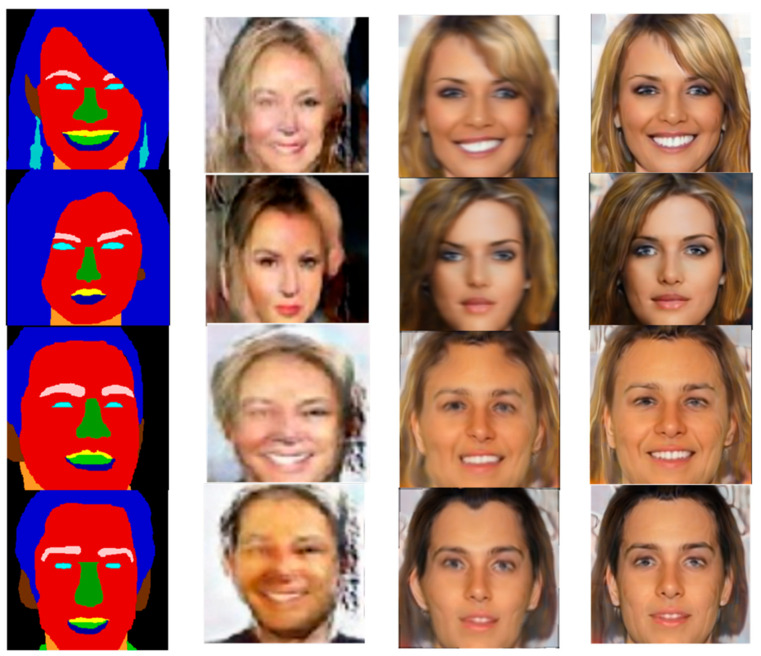
Visualization results of different models on the CelebAMask-HQ dataset.

In order to determine the effectiveness and the optimization effect of the new model, it was compared to other mainstream models. The results from this chapter are compared to PiGAN [9] + StyleGAN [21] and pix2pixHD [30], as shown in Figure 3. Compared to PiGAN + StyleGAN, this new method makes more detailed facial structures and gives better facial feature estimates. Compared to pix2pixHD, the results have clearer facial outlines and edges, with no artifacts on the cheeks.

The performance of the generative model proposed in this paper is evaluated using the FID and IS metrics. As shown in Table 4, the generative model proposed in this paper achieved an FID score of 40.6, indicating a smaller difference between the generated and real images compared to the prediction accuracy of Sem2NeRF, with an improvement of 1.46%. In terms of the IS metric, the model proposed in this chapter obtained a score of 2.15, which indicates that the generated images have greater diversity and a smaller difference compared to real images, with an improvement of 1.90% over the prediction accuracy of Sem2NeRF. While keeping the model parameter size almost unchanged, there is also a certain improvement in the model’s segmentation prediction accuracy, which helps enhance the practicality of the entire project. In addition, NeRFFaceEditing exhibits the lowest FID while minimizing the parameters, indicating that its light consistency and detail fidelity are better than its peers and even better than the slightly larger NeRFNet. Although its IS is significantly higher than that of the baseline model of the conventional GAN, it is slightly lower than that of NeRFNet. FaceLift adopts the latent-diffusion + 3D reconstruction idea, and the number of parameters is about 1.5 times more than that of lightweight NeRFNet, but it fails to outperform NeRFNet in FID/IS. However, compared with large-volume GANs such as pix2pixHD, the “quality-participation” curve is better. When compared with the above two models, it can be seen that the NeRFNet model not only outperforms the traditional GAN model but also outperforms some state-of-the-art models.

**Figure 4:**
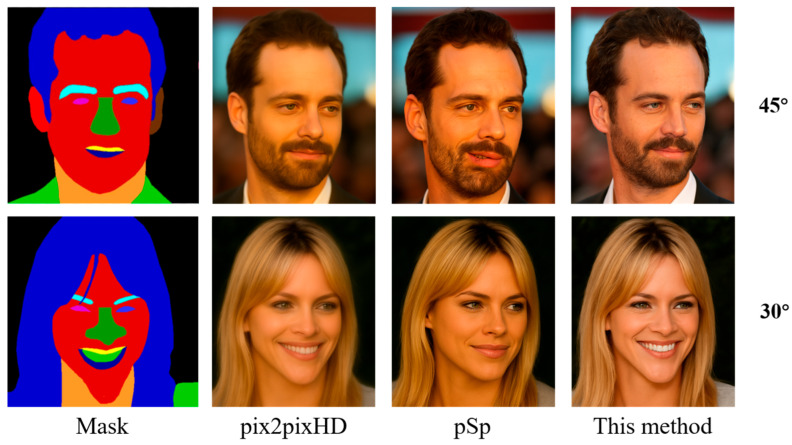
Visualization results of different models on the CelebAMask-HQ dataset.

As shown in Figure 4, the generation results of pix2pixHD, pSp, and the NeRFNet proposed in this paper are shown at 30° and 45° viewing angles, respectively. It can be observed that the image generated by pix2pixHD is of poor quality, with blurred details, less natural skin tones and lighting, and some degree of distortion. The images from pSp show a significant improvement in overall structure and skin color representation but still suffer from a lack of clarity in facial structure and unnatural edge transitions. The images generated by pSp at a 45° viewing angle showed a significant difference in the angle of the upturned corners of the mouth. In addition, the chin length of the image generated by its 30° viewing angle is significantly shorter and does not show the teeth part. The method proposed in this paper, on the other hand, generates the highest quality images with natural facial structure and more realistic lighting and skin color. At the same time, the facial details are rich, especially under the change of face angle, but still maintain a stable sense of realism and consistency. Overall, the method in this paper outperforms existing methods in facial detail restoration, pose adaptation ability, and generation quality and verifies its cross-modal face generation capability under single-view semantic mask conditions.

To confirm the generalization capability of the proposed model, a targeted validation was conducted on the CatMask dataset using FID and IS as the basic evaluation metrics. The quantitative performance information before and after model optimization is shown in Table 5. The overall experiment indicates that, with the model parameter size remaining largely unchanged, the generation performance of the network is superior to that of previous methods, thereby verifying the generalization ability of the proposed approach.

### 4.5. Artistic Effect Evaluation of the Model from the Audience’s Perspective

To ensure that the generated 3D faces are not only technically accurate but also visually appealing and artistically pleasing, a comprehensive evaluation of the model’s artistic effects is conducted. From the 3D face images generated using this model, 25 sets of 3D facial images were selected by stratified random sampling from the model output as a test set for artistic effect assessment.

The 25 sets of evaluation samples covering four types of lighting (natural, sidelight, overhead, and backlight), two types of expressions (basic and complex), and five viewing angles (frontal, 45° lateral, side, overhead, and elevation) were used to examine geometric congruence, muscle deformations, and multi-view light stability. Another three art styles (high-contrast movie style, cartoon style, and retro film style) were added to examine the aesthetic adaptation of non-realistic scenes. Each set of samples is accompanied by 2D semantic masks and technical index labels (SSIM, FID, symmetry parameter, etc.) to facilitate viewers’ comparison of geometric a priori and subjective perception. The test charts were presented in pairs with the Sem2NeRF results, and five sets of randomly inserted CelebAMask-HQ photographs of real people were used as double-blind “realism benchmarks.” All samples were arranged in a Latin square to avoid fatigue, and the weighting of base dimension to style dimension was set to 70%:30% to emphasize the core technical index of the model.

The survey was conducted with 1000 viewers, with the gender ratio set at 45% male and 55% female to balance the difference in attention to facial details. In terms of age distribution, 30% were young adults aged 18–25, primarily college students and young professionals who demonstrate high acceptance of virtual characters and AIGC technology. Middle-aged and young adults aged 26–40 accounted for 50%, including approximately 150 film and television industry practitioners such as directors, visual designers, and special effects artists, as well as general audiences with experience in consuming film and television content. The remaining 20% were middle-aged individuals aged 41–55, mainly traditional film and television creators and senior viewers who prioritize narrative logic and artistic compliance. In terms of professional background, 30% were film and television professionals, focusing on modeling efficiency and artistic control. The other 70% were general audiences with different levels of education, providing aesthetic feedback to the general public.

The generated images were evaluated in terms of four dimensions: realism, aesthetic value, symmetry, and detailed representation. Specific quantitative scores were assigned to each dimension, and final audience ratings were derived from these four dimensions. The fine-grained scoring criteria for the artistic effectiveness evaluation dimensions, which provide descriptive anchors for each scoring band from 1 to 10, are detailed in Table 6.

A score of 1 to 3 represents a serious distortion/imbalance in structure or color scheme and a lack of detail. A score of 4 to 6 means that the face is basically recognizable but with obvious flaws, such as raw lighting, dull composition, symmetry gaps, or a single level. A score of 7 to 8 means that the overall picture is close to reality, with rich and coherent face structure, color, and details, and only slight deficiencies in extreme expressions or special angles. A score of 9 to 10 means that the face is almost identical to a photograph, with highly realistic light, shadow, and materials; professional artistic tension in the composition; symmetry error < 0.3 mm; and vivid and natural micro-textures. Finally, the four scores are combined to form the audience evaluation, which is used to measure the artistic effect of the model.

### 4.6. Artistic Effect Evaluation of the Model

Table 7 shows the results of the experiment conducted by the 1000 viewers mentioned above according to the scoring guidelines in Table 6 and the assessment methods in the previous subsection. The scoring range for each dimension is from 1 to 10, with 1 indicating very poor and 10 indicating excellent.

In terms of realism, the generated 3D faces received a score of 8. This shows that the generated faces look very realistic in terms of lighting and skin texture, especially under different lighting conditions. But there are still a few small issues with details when dealing with complex expressions, like extreme smiles or frowns. These issues take away a little from the overall realism.

In terms of aesthetics, the generated 3D faces scored a 7. They did well in color harmony and consistency, and looked fairly natural overall. However, at some angles, they still looked a little unnatural. This might affect the degree to which viewers feel immersed and their overall enjoyment of the look.

For symmetry, the generated 3D faces achieved a high score of 9. They showed great symmetry in facial features and contours, just like real human faces. This really helped make the faces look more natural and made viewers believe they were realistic.

When it comes to detail representation, the generated 3D faces scored an 8. They did a good job of showing facial texture and details in areas like eyebrows, eyes, nose, and mouth. However, they still need to get better at showing tiny expressions, like the shape of the mouth when smiling, or the lines between the eyebrows when frowning.

Sem2NeRF and pix2pixHD, chosen as comparison models, effectively highlight the strengths of the model presented across multiple critical dimensions. Specifically, the comparison with Sem2NeRF underscores notable advancements of NeRFNet in lighting decoupling and high-quality 3D facial reconstruction. Meanwhile, the contrast with pix2pixHD showcases the superior performance of NeRFNet in single-view generation and 3D effects. Additionally, through audience perception evaluation, the differences in realism, aesthetic value, symmetry, and detail representation among various models are clearly delineated, thereby substantiating the proposed model’s enhanced artistic quality and audience appeal.

The reliability of the statistical results of the assessment scales was further verified in terms of quantitative reliability and between-group differences, respectively, when it was already known that the NeRFNet model had high scale scores. We conducted an internal consistency analysis of viewer ratings calculated for each of the four dimensions. The Cronbach’s alpha coefficient is a statistical measure of the internal consistency reliability of a scale or questionnaire and is used to assess whether a set of question items are concentric in measuring the same attribute on the same underlying dimension. It tells us whether the viewers’ responses on related topics are consistent with each other and therefore reflect the same underlying concept. As can be seen from Table 8, the results of α are all greater than 0.8, which indicates that the scale is reliable.

To verify whether there are inter-group differences between professional practitioners from film and television and general audiences, two independent samples were compared for their statistical results on different assessment dimensions using NeRFNet. First, the two groups of scores were tested for normality and variance alignment. The independent samples *t*-test was performed when normal distribution as well as variance alignment was satisfied. When normal distribution is satisfied but variance is not aligned, the Welch *t*-test is performed. The Mann–Whitney U test is performed when normal distribution is not satisfied.

The validation results are shown in Table 9. It can be seen that the sense of realism and symmetry reached a significant difference in four dimensions, and the rest of the dimensions are *p* greater than 0.05. This indicates that the quality of the images generated by the NeRFNet model can be generally recognized by both the profession and the general public. For realism, t(998) = −3.74, *p* < 0.001, d = 0.24, with a small effect size. This may be due to the fact that the general public is more tolerant of localized imperfections and professionals are more attentive to subtle distortions in extreme expressions. For symmetry, Welch t’(520) = 3.85, *p* < 0.001, d = 0.34, with a medium effect size. This suggests that professional audiences are more sensitive to geometric accuracy and alignment of the five senses, perhaps due to cumulative “occupational sensitivity” from work experience. On aesthetic value, Mann–Whitney U = 100,500, *p* = 0.055, a non-significant difference. It is possible that the difference is only marginally significant due to the fact that film and television professionals are more focused on camera language and stylistic unity. On Detail Representation, Mann–Whitney U = 97,900, *p* = 0.082, a non-significant difference. It is possible that this is due to a convergence in the evaluation of texture and micro-expressions and that the level of detail at this stage meets most people’s expectations. Combined with the high internal consistency of Cronbach’s α > 0.8, the scoring results can be considered reliable. NeRFNet satisfies both industry standards and public aesthetics in terms of multidimensional artistic effects.

Overall, based on the evaluation across the four dimensions, the generated 3D faces demonstrate a high level of artistic effect and visual appeal in the viewing experience. Although there is still room for improvement in certain details and complex expressions, they generally provide a relatively realistic and natural visual experience for the audience. Viewers are likely to recognize this high-quality 3D face generation technology, especially the excellent performance in symmetry and detail representation, which can significantly enhance the immersion and satisfaction of the viewing experience. In the future, further optimization of lighting effects and the naturalness of micro-expressions will help to further improve the artistic effect of the generated faces and the audience’s acceptance.

### 4.7. General Evaluation

The 3D face generation model proposed in this paper, based on the Swin Transformer and NeRF, demonstrates high performance and artistic effects in both objective and subjective evaluations. In terms of objective metrics, the model achieves an excellent score of 40.6 in FID, a 1.46% improvement over other mainstream models such as Sem2NeRF, indicating a higher similarity between the generated face images and real images. The IS reaches 2.15, which is 1.90% higher than the Sem2NeRF model, suggesting that the generated images have greater advantages in terms of diversity and clarity.

In terms of subjective evaluation, the audience gave high ratings to the generated 3D faces across four dimensions: realism, aesthetic value, symmetry consideration, and detail representation. The model particularly excels in symmetry and detail representation, scoring 9.0 and 8.2, respectively. Viewers think the generated faces look very realistic and natural in details like lighting, skin texture, and face shape. However, they also said the tiny expressions and 3D feel need improvement, especially with complex expressions and angles. Overall, the model does an excellent job of making high-quality 3D faces. It still needs more work on handling complex situations better and reaching higher artistic goals.

To correct defects with complex expressions, specific angles, and tiny expression details in the generated 3D faces, some ideas for improvement include adding an Expression-Driven Module. This module can help control expression details better by using an attention mechanism to focus on important areas of expressions, making the 3D faces look more natural in complex expressions. Additionally, a time-series model can be added to capture how expressions change over time, making the transitions between expressions smoother and more natural. Another idea is to include an Angle-Aware Module, which will help the model adjust the 3D look and detail of the generated face based on the viewing angle. This will make the face look more realistic from different angles. Using techniques to boost the 3D feel, the model can make the generated face look more realistic and 3D from the side or at an angle. This will improve how it looks from specific angles. Also, using a multi-scale feature pyramid network can help the model balance the overall face structure and tiny details, making the tiny expressions look better.

## 5. Conclusions

This study introduces an innovative approach that combines a cross-modal generation framework with audience perception evaluation, significantly enhancing the performance of AI-driven 3D face generation in terms of efficiency and artistic expression. By decoupling surface reflectance from lighting conditions and using semantic segmentation masks to map 2D information into 3D space, this method can efficiently generate high-quality 3D face models from single-view inputs. The experimental results show substantial improvements in both technical metrics (SSIM: 0.892, FID: 40.6, IS: 2.15) and artistic expression (symmetry: 9.0, detail representation: 8.2, realism: 8.0, aesthetic value: 7.0). However, further optimization is still needed for the naturalness of complex expressions, the three-dimensionality at specific angles, and the smoothness of dynamic expression transitions.

Sensor technology plays a crucial role in this study. High-precision cameras and depth sensors are used to capture detailed geometric and texture information of the face, while lighting sensors monitor real-time environmental lighting conditions. These rich data sources provide strong support for generating more realistic and natural 3D face models. Future research will focus on improving the naturalness of complex expressions and dynamic performance, leveraging advancements in sensor technology and the integration of multimodal data to further enhance the realism and artistic quality of the generated models.

In summary, this study offers an efficient solution for 3D face generation in the film and television industry, establishing a quantifiable link between technical metrics and artistic effects through audience perception evaluation. As sensor technology continues to advance, the potential applications of AI-generated content in immersive experiences will continue to expand.

In the future, the sensors can be upgraded in order to further improve the geometric accuracy and light consistency. Firstly, a Velodyne VLP-16 LiDAR is introduced to acquire a high-density point cloud of >300 points per square millimeter in real time and implement adaptive NeRF sampling accordingly. It is expected to improve the sampling efficiency by about 30% in critical areas of the face while reducing geometric artifacts. In addition, next-generation ToF depth cameras (e.g., PMD CamCube 3.0) will be evaluated to enhance depth accuracy and noise immunity. In addition, multimodal fusion such as RGB-D and light field can be explored to construct expression-driven a priori that is consistent across frames in time and space. This enhances the model’s realism and artistic expression in dynamic expression rendering.

## Figures and Tables

**Table 1:** Feature extraction network based on Swin Transformer structure.

Input Size	Output Size	Network Layer	c_In	C_Out	N
224 × 224	3136	Path_embed	3	96	1
3136	3136	Dropout	96	96	1
3136	49	basiclayer	96	768	4
49	49	Normlayer	768	768	1
49	1	avgpool	768	768	1
1	-	flatten	768	768	1
-	-	head	768	4608	1

**Table 2:** Ablation experiments with different modules on the CelebAMask-HQ dataset.

Combination	Swin Transformer	NeRF-Based Rendering	Semantic Mask	FID (↓)	IS (↑)
Without ST	×	√	√	44.5	2.07
Without NR	√	×	√	47.2	1.98
Without SM	√	√	×	42.3	2.10
NeRFNet	√	√	√	40.6	2.15

Note: ↓ indicates lower is better; ↑ indicates higher is better.

**Table 3:** Ablation experiments with different loss functions on the CelebAMask-HQ dataset.

Combination	LPIPS Loss	GAN Loss	Pixel-Wise Loss	FID (↓)	IS (↑)
GAN + Pixel-wise Loss	×	√	√	41.8	2.12
LPIPS + Pixel-wise Loss	√	×	√	43.6	2.05
LPIPS + GAN Loss	√	√	×	44.1	2.03
NeRFNet	√	√	√	40.6	2.15

Note: ↓ indicates lower is better; ↑ indicates higher is better.

**Table 4:** Performance comparison of different models on the CelebAMask-HQ dataset.

Models	Parameters (M)	FID (↓)	IS (↑)
π-GAN + StyleGAN [2,20]	148.36	68.2	1.86
pix2pixHD [30]	186.34	46.3	1.77
pSp [31]	140.61	42.1	1.89
Sem2NeRF [32]	34.55	41.2	2.11
NeRFFaceEditing [33]	31.02	40.0	2.10
FaceLift [5]	48.56	44.5	2.06
NeRFNet	35.78	40.6	2.15

Note: ↓ indicates lower is better; ↑ indicates higher is better.

**Table 5:** Performance comparison of different models on the CatMask dataset.

Models	Parameters (M)	FID (↓)	IS (↑)
π-GAN + StyleGAN	148.36	46.2	1.88
pix2pixHD	186.34	30.8	2.33
pSp	140.61	28.6	2.41
Sem2NeRF	34.55	26.2	2.43
NeRFNet	35.78	24.1	2.50

Note: ↓ indicates lower is better; ↑ indicates higher is better.

**Table 6:** Detailed scoring criteria for artistic effect evaluation dimensions.

Evaluation Dimension	1–3	4–6	7–8	9–10
Realism	Unrealistic	Defective	Similar	Perfect
Aesthetic value	Unappealing	Unappealing	Harmonious	High tension
Symmetry	Asymmetric	Defective	Good static	Near-perfect
Detail representation	Missing	Lack variation	Rich	Realistic

**Table 7:** Artistic effect evaluation results of generated 3D faces by viewers.

Evaluation Dimension	Model	Average Score ± SD	Film and Television Practitioners	General Audience	Specific Evaluation
Realism	NeRFNet	8.0 ± 1.2	7.8 ± 1.1	8.1 ± 1.3	High realism with minor flaws in complex expressions.
	Sem2NeRF	7.2 ± 1.3	7.0 ± 1.2	7.4 ± 1.4	Good realism, issues in complex expressions.
	pix2pixHD	6.8 ± 1.4	6.5 ± 1.3	7.1 ± 1.5	Basic realism, lacks detail.
Aesthetic Value	NeRFNet	7.0 ± 1.5	6.5 ± 1.4	7.2 ± 1.6	Harmonious colors, natural overall.
	Sem2NeRF	6.5 ± 1.6	6.0 ± 1.5	7.0 ± 1.7	Decent aesthetics, lacks appeal.
	pix2pixHD	6.2 ± 1.7	5.8 ± 1.6	6.6 ± 1.8	Basic aesthetics, inconsistent.
Symmetry	NeRFNet	9.0 ± 0.8	9.2 ± 0.7	8.9 ± 0.9	High symmetry, very natural.
	Sem2NeRF	8.5 ± 0.9	8.3 ± 0.8	8.7 ± 1.0	Good symmetry, slight asymmetry in dynamic expressions.
	pix2pixHD	8.2 ± 1.0	8.0 ± 1.1	8.4 ± 1.2	Basic symmetry, less consistent.
Detail Representation	NeRFNet	8.2 ± 1.0	8.5 ± 0.9	8.1 ± 1.1	Good detail, needs refinement in micro-expressions.
	Sem2NeRF	7.5 ± 1.1	7.2 ± 1.2	7.8 ± 1.3	Decent detail, lacks refinement.
	pix2pixHD	7.0 ± 1.2	6.8 ± 1.3	7.2 ± 1.4	Basic detail, lacks depth.

**Table 8:** Internal consistency of viewer ratings (Cronbach’s alpha).

Evaluation Dimension	α
Realism	0.85
Aesthetic Value	0.82
Symmetry	0.89
Detail Representation	0.84

**Table 9:** Statistical analysis of differences in audience groups (Film and Television Practitioners vs. General Audience).

Evaluation Dimension	Film and Television Practitioners (n = 300)	General Audience (n = 700)	Test Methods	Test Statistic	*p*-Value
Realism	7.8 ± 1.1	8.1 ± 1.3	*t*-test	t(998) = −3.74	*p* < 0.001, d = 0.24
Aesthetic Value	6.5 ± 1.4	7.2 ± 1.6	Mann–Whitney	U = 100,500	0.055
Symmetry	9.2 ± 0.7	8.9 ± 0.9	Welch t	t’(520) = 3.85	*p* < 0.001, d = 0.34
Detail Representation	8.5 ± 0.9	8.1 ± 1.1	Mann–Whitney	U = 97,900	0.082

## Data Availability

All data generated or analyzed during this study are included in this article. The raw data are available from the corresponding author upon reasonable request.
